# Nitrates and Digitalis in Angina Pectoris

**Published:** 1911-03

**Authors:** 


### Nitrates and Digitalis in Angina Pectoris

HERRICK in the *Journal of the American Medical Association* of October 22, 1910, refers briefly to certain fallacies concerning the use of nitrites and digitalis.

The nitrites have, especially of late, been held by some in less repute than formerly, because they fail completely in some instances to relieve or ward off the attacks. Others seem so convinced of their efficacy that, when in a case of supposed angina the nitrites fail to relieve the suffering, and to relieve it promptly, they conclude that they are dealing with something different from true angina. Now, the truth seems to be that nitrites in some cases give most prompt, marvelously prompt, relief. The testimony of many physicians is available on this point. It is hard to understand, then, how some good men—Romberg, for example—regard this remedy as either of no benefit or of very insignificant value, at least during a seizure. Unless convinced by repeated trials as to its usefulness in the attacks, no patient should fail to keep in his pocket either the pearls of nitrite of amyl or the pill or tablet of nitroglycerin, grain 1|100 to 1|50. Some attacks are surely cut short by the use of these remedies, and often the patient has a slight warning of the onset, and the timely use of the drug may prevent a paroxysm.

No matter how strong the prejudice against the use of digitalis in cardiac hypertrophy and general arteriosclerosis may be, no matter what may have been said against its employment in weakened myocardial states, there can be no question that in some cases of angina—not easily described—this drug does more good than all the iodides or nitrites.

Herrick's views may be summarised by making the statement as a series of negations:

1. With "truth," organic cardiovascular angina pectoris there cannot always be made out peripheral arteriosclerosis, cardiac hypertrophy, and high blood-pressure.

2. The finding of aneurism or lesion of the aortic valves does not exclude angina, but rather argues in its favor.

3. The attacks are not always few in number and brief in duration; they do not occur solely after exertion.

4. While the prognosis is grave, life is not necessarily limited to a few months.

5. The disease is by no means to be excluded because the symptoms are found in a woman, even in a nervous or hysterical woman.

6. The pain is not necessarily severe; it may be mild or even lacking; its radiation is variable.

7. The statement that the patient is always conscious, that he is always immobile and erect, must be modified so as to read "usually" instead of "always."

8. Eructations or vomiting during an attack do not argue conclusively against the cardiovascular origin of the angina.

9. It is not correct to look on the nitrites as of no benefit in relieving symptoms; nor, on the contrary, is one justified in concluding that a case unrelieved by nitrites is not angina.

10. Digitalis is not necessarily contra-indicated; it is often of very great value.

Closer observation of the atypical as well as the typical cases, with collocation of the results with those of anatomical and experimental studies, will lead to unifying knowledge concerning the pathogenesis of this disease, to greater precision in its recognition, and to more appropriate treatment.—*Therapeutic Gazette.*

